# Sustained spike-specific IgG antibodies following CoronaVac (Sinovac) vaccination in sub-Saharan Africa, but increased breakthrough infections in baseline spike-naive individuals

**DOI:** 10.3389/fimmu.2023.1255676

**Published:** 2023-11-30

**Authors:** Jackson Sembera, Claire Baine, Violet Ankunda, Joseph Ssebwana Katende, Gerald Kevin Oluka, Christine Hermilia Akoli, Laban Kato, Geoffrey Odoch, Peter Ejou, Solomon Opio, Monica Musenero, The COVID-19 Immunoprofiling Team, Pontiano Kaleebu, Jennifer Serwanga

**Affiliations:** ^1^ Department of Immunology, Uganda Virus Research Institute, Entebbe, Uganda; ^2^ Pathogen Genomics, Phenotype, and Immunity Program, Medical Research Council, Uganda Virus Research Institute and London School of Hygiene and Tropical Medicine, Uganda Research Unit, Entebbe, Uganda; ^3^ Science, Technology, and Innovation Secretariat, Office of the President, Government of Uganda, Kampala, Uganda

**Keywords:** CoronaVac, COVID-19, antibody concentrations, seropositivity, antibody persistence, breakthrough infections, spike protein, sub-Saharan Africa

## Abstract

**Introduction:**

This study investigated the antibody responses to the inactivated COVID-19 vaccine, CoronaVac (Sinovac Biotech) in the African population to provide valuable insights into long-term immunity and breakthrough infections against SARS-CoV-2 in individuals with varying prior IgG seropositivity.

**Methods:**

Real-life cohorts were used to longitudinally track antibody levels against the SARS-CoV-2 spike and nucleoprotein in 60 participants over 12 months to examine the levels of multiple antibody isotypes (S-IgG, S-IgM, S-IgA, N-IgG, and N-IgM).

**Results:**

Throughout the 12 months, we observed consistently high and stable seropositivity rates for spike-IgG antibodies, spike-IgM antibodies showed a decline in frequencies over time, and spike-IgA levels remained moderate and stable. Vaccinated individuals previously positive for spike-IgG antibodies demonstrated strong and persistent seropositivity, while those initially negative experienced a gradual and delayed increase in seropositivity rates. The fold change analysis of S- and N- antibody responses demonstrated a consistently stable and comparable profile over time, indicating that vaccine-induced antibody responses remain constant and lack significant fluctuations beyond the initial boost. The study emphasized that individuals lacking previous IgG positivity showed reduced vaccine-induced spike-IgG antibodies and were more susceptible to breakthrough infections, highlighting their higher vulnerability. All cases of breakthrough infections were asymptomatic, indicating the conferred protection to the vaccinated individuals.

**Discussion:**

The findings corroborated earlier studies on the effectiveness of the CoronaVac vaccine and emphasized the significance of accounting for pre-existing seropositivity in vaccine assessments. This study effectively demonstrated durable antibody responses against SARS-CoV-2 in the African population following the CoronaVac vaccination, providing crucial insights for informing vaccination strategies and safeguarding vulnerable populations. Continuous surveillance is imperative for tracking breakthrough infections and monitoring waning immunity. The insights gained offer crucial direction for public health strategies and enhance comprehension of vaccine effectiveness in sub-Saharan Africa. Further research should explore functional outcomes, cellular immune responses, and the vaccine’s effectiveness against different variants to enhance our understanding and optimize vaccine strategies.

## Introduction

The global fight against infectious viral diseases heavily relies on the widespread use of vaccines (1, 2), which stimulate the immune system to combat specific pathogens. In recent years, the world faced an unprecedented challenge with the Coronavirus Disease 2019 (COVID-19) pandemic caused by the novel severe acute respiratory syndrome coronavirus 2 (SARS-CoV-2). To combat this highly contagious and potentially deadly virus, various vaccines were developed and deployed worldwide (3). The inactivated COVID-19 vaccine, CoronaVac, developed by Sinovac Biotech Ltd. and distributed through the COVID-19 Vaccines Global Access (COVAX) facility, gained significant prominence in sub-Saharan Africa (SSA) as one of the region’s primary COVID-19 vaccines. CoronaVac was widely administered in SSA and other settings, including China, Brazil, and Turkey. In those settings, CoronaVac showed lower antibody concentrations than other vaccines (4), a robust but transient peak in IgG levels (3), more durable immune responses in individuals with prior infection from the virus (5), and persistence of humoral and cell-mediated immunity till 12 months post-vaccination, despite declining antibody titers (6).

Despite its extensive regional distribution in SSA (4), limited data exist on the population’s response to the CoronaVac vaccine. Understanding the performance of CoronaVac in the sub-Saharan African context is essential for formulating effective vaccination strategies, informing public health decisions, and maximizing the overall effectiveness of vaccination campaigns in the region. Evaluating immune responses to CoronaVac in SSA is vital for maximizing vaccination efforts by tailoring interventions to the specific characteristics of the local population and accounting for regional variations in immune traits, genetic diversity, and coexisting health conditions that may affect the effectiveness of vaccination (7–9).

Accurate data on CoronaVac’s performance in sub-Saharan Africa is vital for informing policymakers and healthcare professionals in designing tailored immunization programs considering the region’s resource constraints and unique healthcare challenges. This study seeks to assess the antibody responses to CoronaVac in the SSA population. It evaluates the vaccine’s generation of spike and nucleoprotein-specific IgG, IgM, and IgA antibodies and examines their longevity in real-world settings.

## Materials and methods

### Study population

Over 12 months, from November 25th, 2021, to May 5th, 2023, 423 blood samples were obtained from 60 individuals who had received both doses of the inactivated COVID-19 vaccine, CoronaVac. A total of 423 samples were collected from 60 participants between November 25, 2021, and May 5, 2023. The participants included 23 females (38.3%) and 37 males (61.7%), with ages ranging from 18 to 47 years and a median age of 22.0 years, interquartile range (20.0–24.3) years. Samples were collected to evaluate the immediate effects of the vaccine 14 days (D14PP, n=54) and 28 days (D28PP, n=54) after the initial dose. Samples were collected at two additional time points, 14 days (D14PP), and 28 days (D28PP) after the second dose, from 49 participants. Additional samples were collected at six months (M6PP, n = 54), nine months (M9PP, n = 52), and twelve months (M12PP, n = 51) post-initial vaccination to assess the vaccine’s long-term effects. The study protocols received ethical approval from the Uganda Virus Research Institute (UVRI REC Ref: GC/127/20/04/773) and the Uganda National Council for Science and Technology (UNCST Ref: HS637ES). All study participants provided written informed consent before participation.

### Binding antibody ELISA to detect SARS-CoV-2-specific IgG, IgM, and IgA levels

To ascertain the presence and concentrations of SARS-CoV-2-specific antibodies, we used a binding antibody enzyme-linked immunosorbent assay (ELISA) previously validated and optimized for this population. This assay detected the optical densities at 450 nm (OD at 450nm) and the corresponding IgG, IgM, and IgA concentrations, expressed in ng/ml, specific to the spike and nucleoprotein antigens. The comprehensive ELISA procedure was previously validated in this population (10) and described in detail elsewhere (11).

### Statistical methods

Box plots were used to compare the data’s medians, means, and quartiles. Horizontal line graphs illustrated each participant’s seroconversion over the entire study duration. Line plots were used to present the percentages of seropositive individuals. A Wilcoxon Rank sum test was employed for pairwise comparisons to assess the significance of differences in antibody responses between different time points. To account for multiple testing, a Bonferroni correction was applied. Since there were missing data/samples at various time points, unpaired tests were considered. In the statistical analysis, a p-value greater than 0.05 was deemed insignificant (indicated as “ns”). Statistical significance was denoted using asterisks: * represented a p-value less than 0.05, ** denoted a p-value less than 0.01, and *** indicated a p-value less than 0.001.

## Results

### S-IgG positive individuals exhibit early moderate responses with a subsequent decline and rebound, while negative individuals equilibrate by day 28 post-prime

A marked difference was observed in S-IgG OD and concentration between the positive and negative groups up to 28 days after boosting. From this point, the distribution between the two groups became indistinguishable. Those testing positive for S-IgG initially exhibited moderate antibody responses, which remained stable up to six months post-prime, after which they began to decline, only to rise again at nine months, possibly due to re-infection. In contrast, the S-IgG negative group started with considerably lower antibody levels, aligning with the levels seen in the positive group by 28 days post-prime. This group also experienced a decline after six months but witnessed an increase 12 months later.

There were no significant differences between the positive and negative groups across all time points for S-IgM antibody responses. S-IgA responses, however, varied between the two groups. The positive S-IgG group had S-IgA levels slightly above the cut-off and remained stable until M6PP, whereas the S-IgG negative group had values below the cut-off. By six and nine after priming, there was no discernible difference in S-IgA responses between the groups.

**Figure 1:**
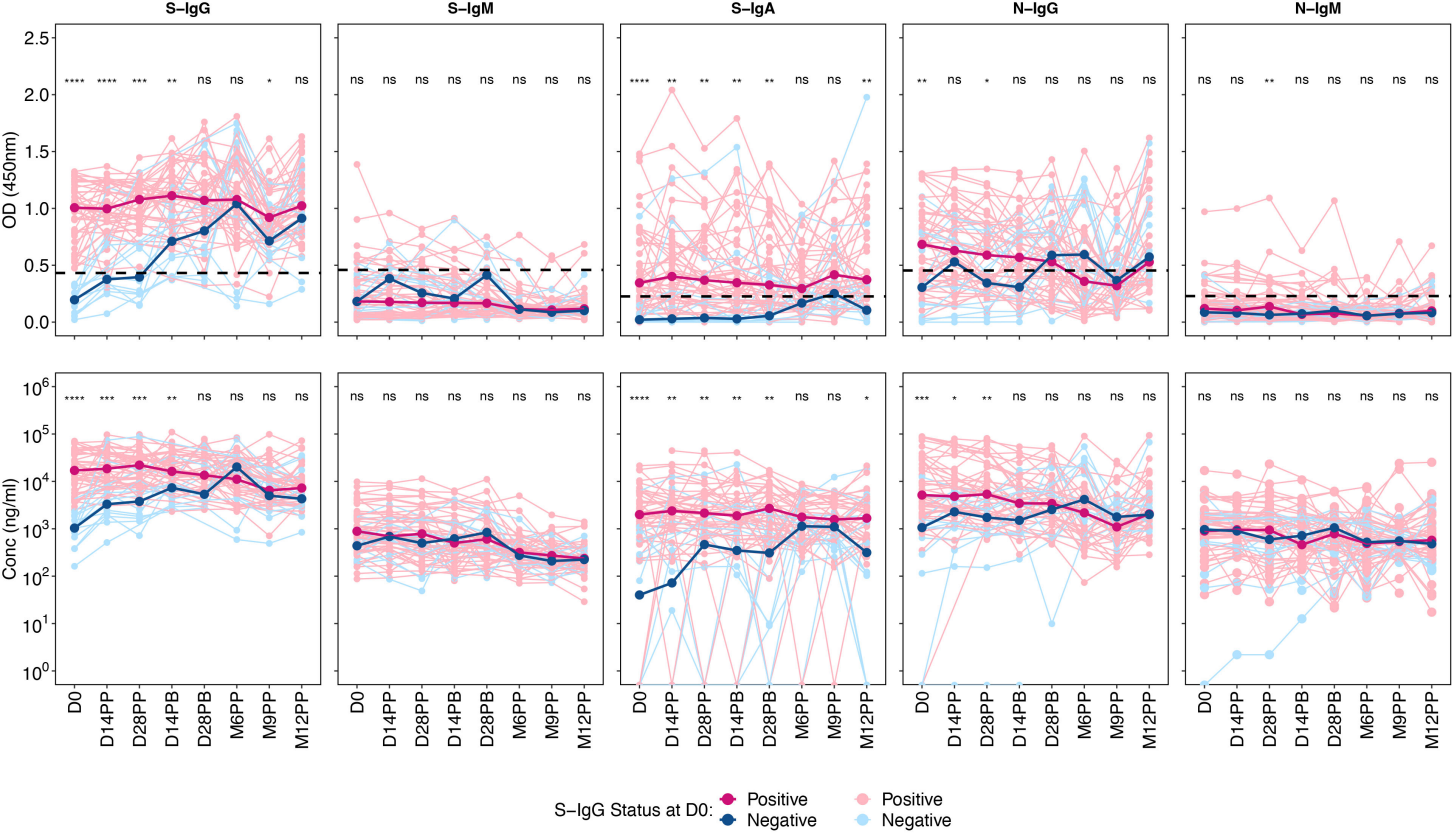
Optical Densities and Concentrations of Spike- and Nucleocapsid-Directed Antibodies Following CoronaVac Vaccination, Stratified by Baseline S-IgG Levels. **Figure 1** presents the optical densities (OD) and concentrations of Spike (S) and Nucleocapsid (N) proteins, segmented by subjects with baseline S-IgG positivity and negativity. An unmatched samples Wilcoxon test was employed to assess the differences in OD or concentration between the positive (red) and negative (blue) subjects at specific post-vaccination intervals. The thin, lighter lines represent individual profiles, whereas the bolder, darker lines signify the group’s median. A p-value greater than 0.05 was deemed insignificant (indicated as “ns”; not significant). Statistical significance is denoted by “*” representing a p-value less than 0.05, “**” for a p-value less than 0.01, “***” indicates a p-value less than 0.001, while “****” indicates a p-value less than 0.0001.

Regarding N-IgG responses, the positive group consistently showed declining yet higher levels than the negative group at the onset. The trend for the positive group continued to decline until M9PP, followed by an increase. The negative group, starting with notably lower N-IgG responses, saw an increase that, by 14 days post-prime, nearly matched the levels in the positive group. Lastly, both groups exhibited low and comparable N-IgM responses, with no significant difference detected, summarized in **Figure 1**.

### From the baseline onward, we observed a notable degree of cross-reactivity

**Figure 2:**
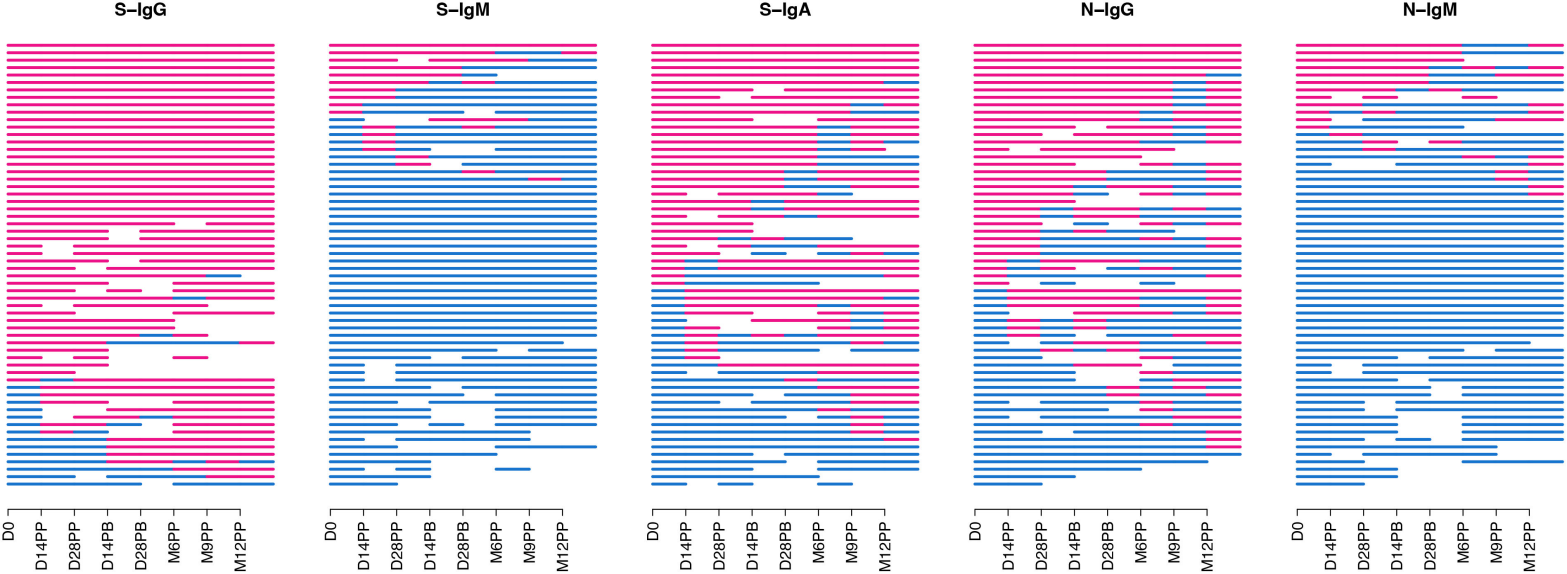
Seropositivity of S-and N-directed Antibodies Following CoronaVac Vaccination. **Figure 2** summarizes the temporal seroconversion patterns of participants. The horizontal line graphs represent the seropositivity status of each participant at various time points. Each line symbolizes an individual, tracing their shift between positive serostatus (OD antibody response above cut-off, in red) and negative serostatus (OD response below cut-off, in blue).

A consistently high frequency of seropositivity for spike-IgG antibodies was observed throughout the follow-up period (**Figure 2**), with 76.7% of the cohort being seropositive at baseline. Seropositivity levels reached 87% before the second dose administration. They remained stable for 12 months, demonstrating a persistent and resilient binding antibody response with consistently high levels of S-IgG antibodies throughout the study period. There was a consistent decline in S-IgM antibody frequencies, from 16.7% at baseline to 13% after the second dose and reaching 3.9% at 12 months, indicating suboptimal levels of IgM antibody responses with limited detectable responders during the follow-up period. Spike-IgA antibody levels showed a moderate increase, with seropositivity rising from a baseline of 55% to 61% before the second dose and maintaining a steady frequency of 64.7% at the 12-month mark. Overall, the CoronaVac vaccine demonstrated sustained IgG antibody seropositivity in individuals previously exposed to the spike protein.

In the overall cohort, horizontal nucleoprotein line graphs consistently demonstrated seropositivity of N-IgG in most participants, persisting for up to six months of the study. The initial N-IgG positivity rate of 55% demonstrated substantial prior exposure, slightly decreasing to 46.3% at six months post-primary vaccination, demonstrating continued nucleoprotein cross-reactivity. However, at nine months, the rate dropped to 25%, followed by a significant rise to 64.7% at twelve months, suggesting the possibility of infection. Baseline IgM antibody levels were initially low at 20%, with a subsequent gradual decline to 5.6% and 7.7% at six and nine months, respectively Breakthrough infections refer to SARS-CoV-2 infections that occur within two weeks after the completion of a two-dose vaccination and are characterized by a minimum 11-fold rise in N-IgG concentration. Out of the 60 individuals who received the CoronaVac vaccine, 11 cases of breakthrough infections were detected, indicating an incidence rate of 18.3%; notably, one participant experienced two breakthrough infections 14 days after complete vaccination and again after 12 months. An additional participant tested positive for the infection four weeks after receiving the booster shot, while three individuals contracted the infection after six months, one after nine months, and five individuals within the one year following vaccination. The study reveals presumed breakthrough infections in individuals who have received full vaccination.

### Anti-spike antibody concentrations showed stable IgG, moderate IgA, and low IgM levels

The World Health Organization (WHO) classifies anti-SARS-CoV-2 antibody titers within the ranges of 44-53 BAU/mL as low, 200-300 BAU/mL as moderate, and 700-800 BAU/mL as high (10, 12, 13). The cohort’s anti-spike IgG antibody levels were evaluated per the World Health Organization (WHO) guidelines. Assessments of overall antibody concentrations utilized means, medians, and interquartile ranges (IQR), demonstrating consistently sustained S-IgG antibodies with no significant difference between consecutive time points. Overall, the median concentrations of S-IgG displayed consistent moderate levels throughout the follow-up period of 12 months, ranging from 207 (65-565) at baseline to 268 at day 28 post-prime and then gradually decreasing by half to 118 (70-241) and 131 (68-202) BAU/ml at 9- and 12-months post-protocol, respectively. The observed results demonstrate a consistent and robust immune response in the participants. The median antibody levels of 247.2 BAU/ml (IQR 227.9 - 268.0 BAU/ml) correspond to the WHO’s classification of moderate antibody levels, indicating a sustained and moderate immune response (**Table 1**). The reduced levels corresponded to about half of the previous measurements and were observed concurrently with breakthrough infections.

**Table 1:** presents the temporal evolution of antibody optical densities (OD) and concentrations in 60 individuals after vaccination with the CoronaVac vaccine.

Time Point	Antibody	Median OD (IQR) (450nm)	Median Conc (IQR) (ng/ml)	Median Conc (IQR) (BAU/ml)
D0	S-IgG	0.900 (0.476, 1.141)	11058.4 (3469.45, 30180.93)	207.193 (65.060, 565.339)
	S-IgM	0.182 (0.059, 0.377)	738.750 (364.575, 1666.075)	27.7228 (13.917, 61.9383)
	S-IgA	0.289 (0.074, 0.537)	1872.60 (840.875, 4850.200)	357.352 (160.45, 925.630)
	N-IgG	0.554 (0.306, 0.824)	4056.20 (1700.48, 14744.73)	41.4352 (17.467, 159.907)
D14PP	S-IgG	0.948 (0.721, 1.148)	12534.8 (6610.50, 34750.60)	234.845 (123.89, 650.924)
	S-IgM	0.222 (0.078, 0.412)	684.400 (408.750, 1714.975)	25.7174 (15.547, 63.7426)
	S-IgA	0.320 (0.125, 0.628)	2297.05 (964.600, 4139.200)	438.358 (184.06, 789.935)
	N-IgG	0.630 (0.345, 0.896)	4133.50 (2377.65, 10541.73)	47.7373 (27.593, 122.351)
D28PP	S-IgG	0.965 (0.780, 1.175)	14300.0 (6995.50, 31515.50)	267.905 (131.10, 590.334)
	S-IgM	0.174 (0.068, 0.388)	700.800 (311.100, 1617.000)	26.3225 (11.944, 60.1276)
	S-IgA	0.301 (0.155, 0.604)	2105.25 (1012.85, 5385.100)	401.753 (193.27, 1027.72)
	N-IgG	0.505 (0.297, 0.849)	4346.50 (1712.30, 10004.40)	48.7480 (18.771, 116.501)
D14PB	S-IgG	1.036 (0.779, 1.227)	13511.5 (7245.90, 30514.60)	253.137 (135.79, 571.588)
	S-IgM	0.184 (0.101, 0.333)	546.000 (247.100, 1343.900)	20.6109 (9.5823, 50.0510)
	S-IgA	0.260 (0.104, 0.548)	1717.68 (860.650, 3594.800)	327.784 (164.22, 686.035)
	N-IgG	0.546 (0.300, 0.791)	3181.55 (1381.18, 8332.275)	36.8016 (16.071, 95.5949)
D28PB	S-IgG	1.042 (0.773, 1.233)	12879.1 (5565.90, 30871.74)	241.293 (104.32, 578.277)
	S-IgM	0.176 (0.093, 0.368)	652.000 (284.200, 1683.200)	24.5220 (10.951, 62.5702)
	S-IgA	0.264 (0.119, 0.543)	1634.63 (579.825, 4967.850)	311.934 (110.62, 948.083)
	N-IgG	0.533 (0.274, 0.782)	3118.70 (1632.10, 6673.075)	36.5884 (19.268, 78.0005)
M6PP	S-IgG	1.053 (0.863, 1.421)	14318.0 (5056.70, 31017.52)	268.243 (94.787, 581.008)
	S-IgM	0.113 (0.070, 0.157)	299.800 (205.250, 501.1750)	11.5268 (8.0382, 18.9570)
	S-IgA	0.279 (0.110, 0.499)	1508.35 (536.175, 3410.150)	287.834 (102.29, 650.795)
	N-IgG	0.429 (0.163, 0.740)	2428.50 (737.100, 5552.000)	28.5469 (8.8404, 64.9388)
M9PP	S-IgG	0.846 (0.699, 1.004)	6313.80 (3723.65, 12883.52)	118.331 (69.821, 241.376)
	S-IgM	0.102 (0.073, 0.137)	255.850 (163.750, 387.3250)	9.90521 (6.5070, 14.7563)
	S-IgA	0.345 (0.190, 0.633)	1564.10 (765.000, 3186.050)	298.474 (145.97, 608.025)
	N-IgG	0.332 (0.200, 0.459)	1328.35 (638.850, 2529.550)	15.7291 (7.6957, 29.7242)
M12PP	S-IgG	0.991 (0.815, 1.205)	6973.40 (3619.85, 10778.00)	130.685 (67.877, 201.942)
	S-IgM	0.114 (0.075, 0.163)	229.800 (149.500, 325.4000)	8.94405 (5.9812, 12.4714)
	S-IgA	0.336 (0.169, 0.766)	1605.50 (612.700, 3644.150)	306.375 (116.90, 695.454)
	N-IgG	0.538 (0.376, 0.980)	2064.60 (1266.00, 5211.250)	24.3071 (15.003, 60.9687)

We found a continuous decrease in S-IgM levels throughout the study. The median concentration was 26 BAU/ml (IQR: 23-27 BAU/ml), and notable declines were observed at M6PP, M9PP, and M12PP, with levels dropping to 11.5, 9.9, and 8.9 BAU/ml, respectively. A stable and moderate range of S-IgA responses, ranging from 241 to 438 BAU/ml, was consistently observed with no significant variations across different time points. The nucleoprotein elicited consistently moderate levels of IgG throughout, while the concentrations of IgM remained consistently low, with no notable variation observed across the evaluated time points. The data demonstrate persistent S-IgG antibodies, while S-IgM levels remained consistently low. Furthermore, there were moderate levels of S-IgA antibodies, and the concentrations of N-IgG remained stable throughout the observed period.

**Figure 3:**
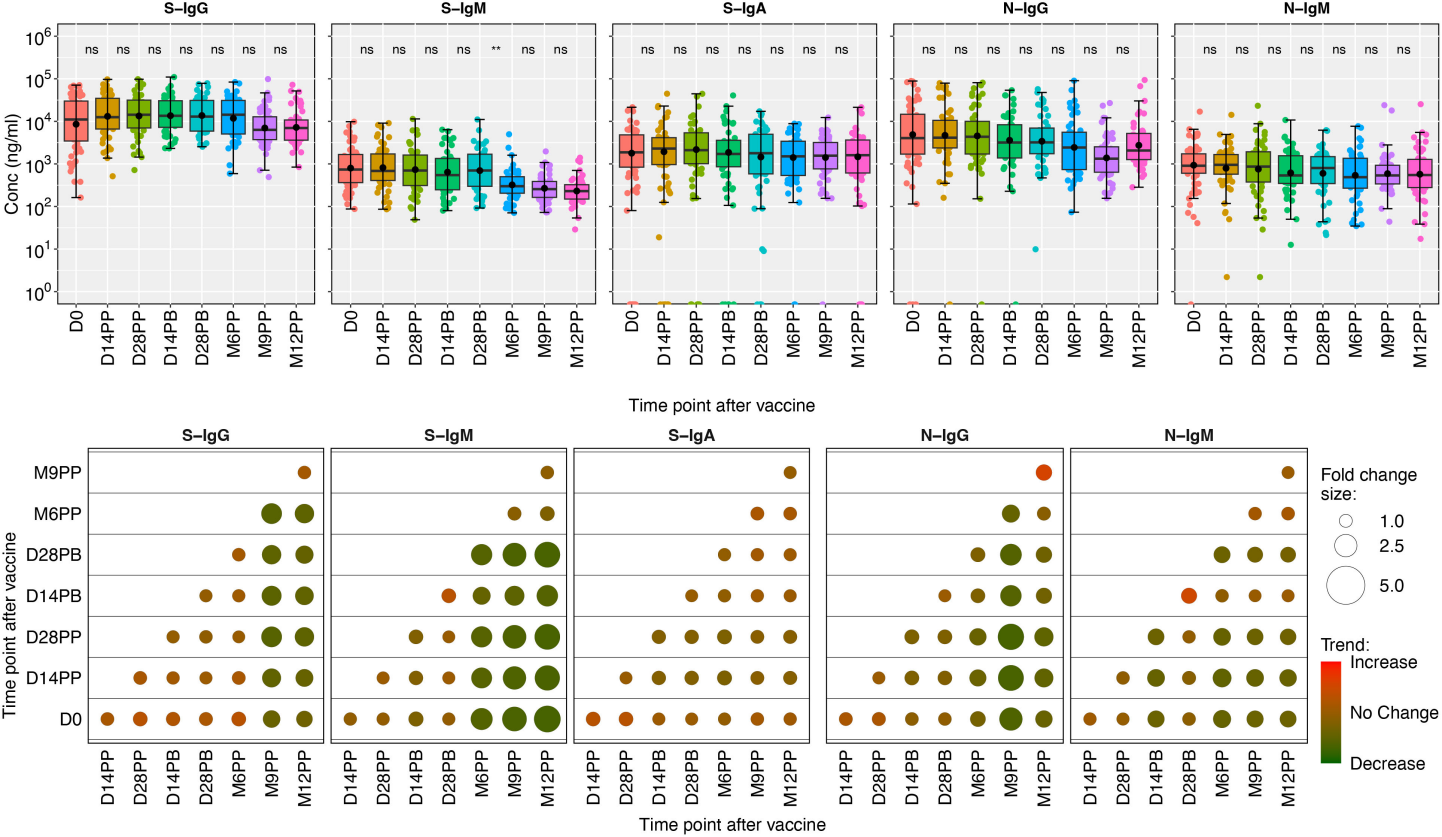
Boxplots illustrating the concentrations of S-and N-directed antibodies and their corresponding fold changes. **Figure 3** shows box plots representing the mean values (dots), medians (horizontal lines), and interquartile ranges (top and bottom of the box) for spike- and nucleoprotein-directed IgG, IgM, and IgA antibody concentrations over time. The x-axis represents the timeline for specimen evaluations, beginning with the baseline (D0), followed by 14- and 28-days post-prime (14PP and 28PP), 28 days post-boost (28PB), and then at intervals of 6-, 9-, and 12-months post-prime (M6PP, M9PP, and M12PP). The y-axis represents concentrations of the respective antibodies in mg/ml Corresponding fold changes in antibody concentrations are visually presented as median fold changes between consecutive time points, with an increase in red shading and a decrease in green shading. The size of the circle corresponds to the magnitude of the change. Insignificance is denoted by p-values greater than 0.05, represented as “ns” (ns; not significant). Statistical significance is indicated by ** (p < 0.01).

Median fold changes between each pair of time points were graphically displayed using color shading, where red represented increases, green represented decreases, and the size of the circles in the plots corresponded to the magnitude of the observed changes. The analysis showed slight increases in IgG and IgA responses against the spike and noticeable decreases in S-IgM levels. The longitudinal analysis demonstrated minor changes in antibodies targeting nucleoprotein, namely N-IgG and N-IgM, except for a visually discernible surge in median N-IgG responses observed between M9PP and M12PP, as depicted by a deeper red shade compared to other time points. Throughout the study, the fold change between pairwise time points consistently approached 1, suggesting a consistent and comparable level of S- and N- antibody responses. These data are summarized in **Figure 3**.

### Persistent S-IgG, suboptimal S-IgM, and low S-IgA levels in previously exposed individuals after CoronaVac vaccination: implications for reinfection risk

**Figure 4:**
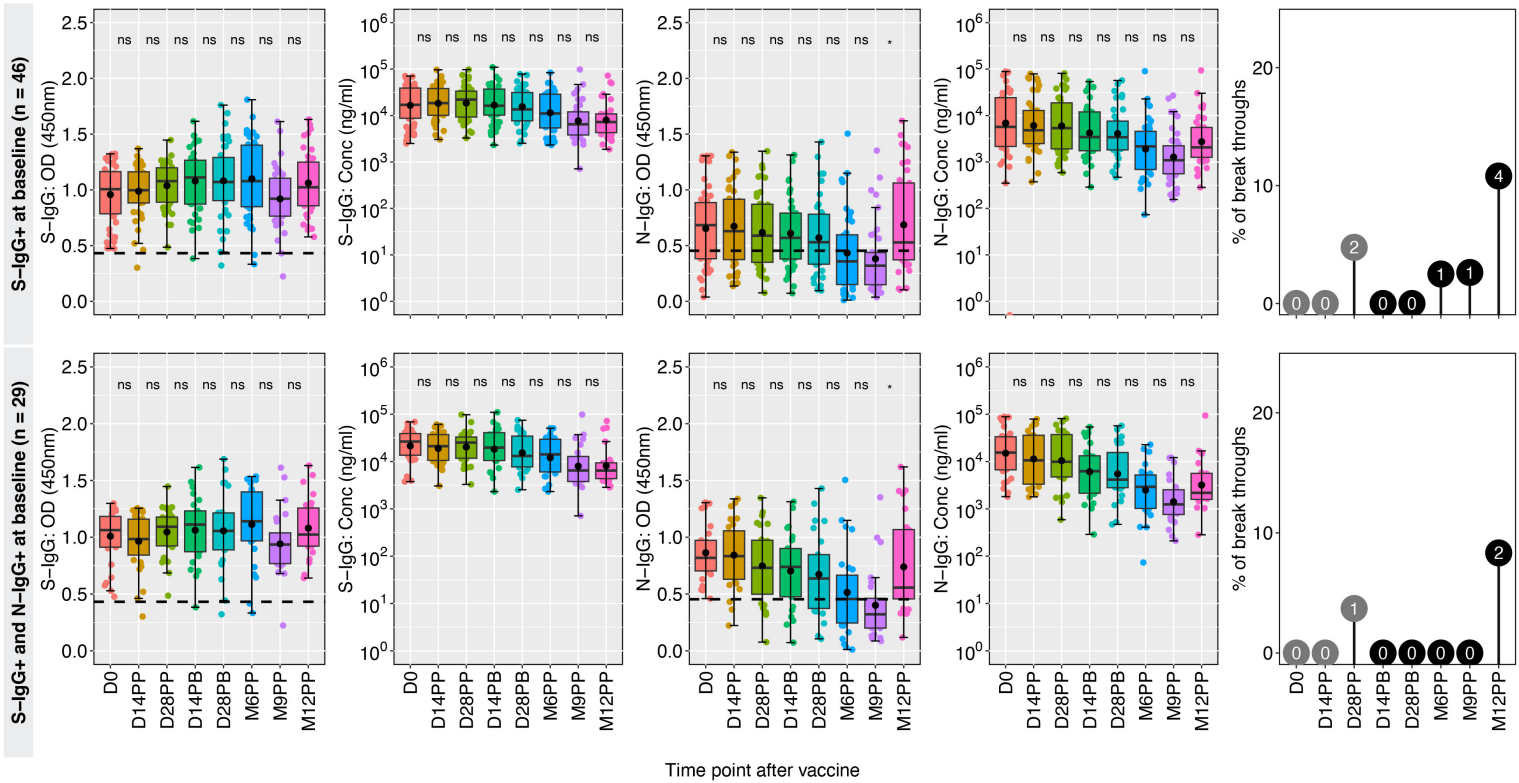
Hybrid immunity among the previously exposed group. **Figure 4** illustrates the dynamics of antibody responses at various time points in a cohort of 46 individuals who were seropositive for S-IgG+ and 26 individuals with combined seropositivity for S-IgG+ and N-IgG+ at baseline (D0), indicating prior exposure before vaccination. Box plots represent the summarized data of spike-directed IgG antibody OD values and concentrations, including the means (represented by dots), medians (horizontal lines), and interquartile ranges (top and bottom of the box). Additional boxplots display the OD values for S-IgA and N-IgG antibodies. Infections before the full vaccine dose are highlighted as numbers inside grey circles, while breakthroughs defined as infections within two weeks of completing the full dose are indicated as numbers inside black circles. The values enclosed in circles represent absolute numbers. The number of infections is indicated within the corresponding circles, providing insights into the infection patterns and vaccine efficacy. “*” represents a p-value less than 0.05; ns, not significant.

The spike-directed IgG positivity levels fluctuated, correlating with the subjects’ baseline prior S-IgG cross reactivity. Using baseline IgG, the 60 participants were categorized as either previously exposed (46 positive S-IgG) or unexposed (14 negative S-IgG). Sustained seropositivity and robust S-IgG antibody concentrations were observed for the 46 previously exposed participants, while their S-IgA antibody levels decreased. Concurrently, N-IgG responses exhibited a modest initial baseline, declined gradually, reaching suboptimal after six months, then significantly increased at 12 months, indicating potential reinfection. Thirteen per cent of the 46 spike seropositive individuals at baseline experienced breakthrough infections, with the initial infection occurring six months after receiving the primary dose, these data are summarized in **Figure 4**.

Hybrid immunity likely accounts for the consistently high and comparable S-IgG antibody levels, resulting in a 93% to 100% seropositivity rate among the 29 participants with baseline seropositivity for S-IgG and N-IgG. The participants initially had low levels of S-IgM and S-IgA, with N-IgG responses decreasing until nine months after vaccination, followed by a significant rise. Monitoring long-term antibody response dynamics is crucial for understanding reinfection occurrences.

### Higher breakthrough rates in baseline spike antibody-naive participants

**Figure 5:**
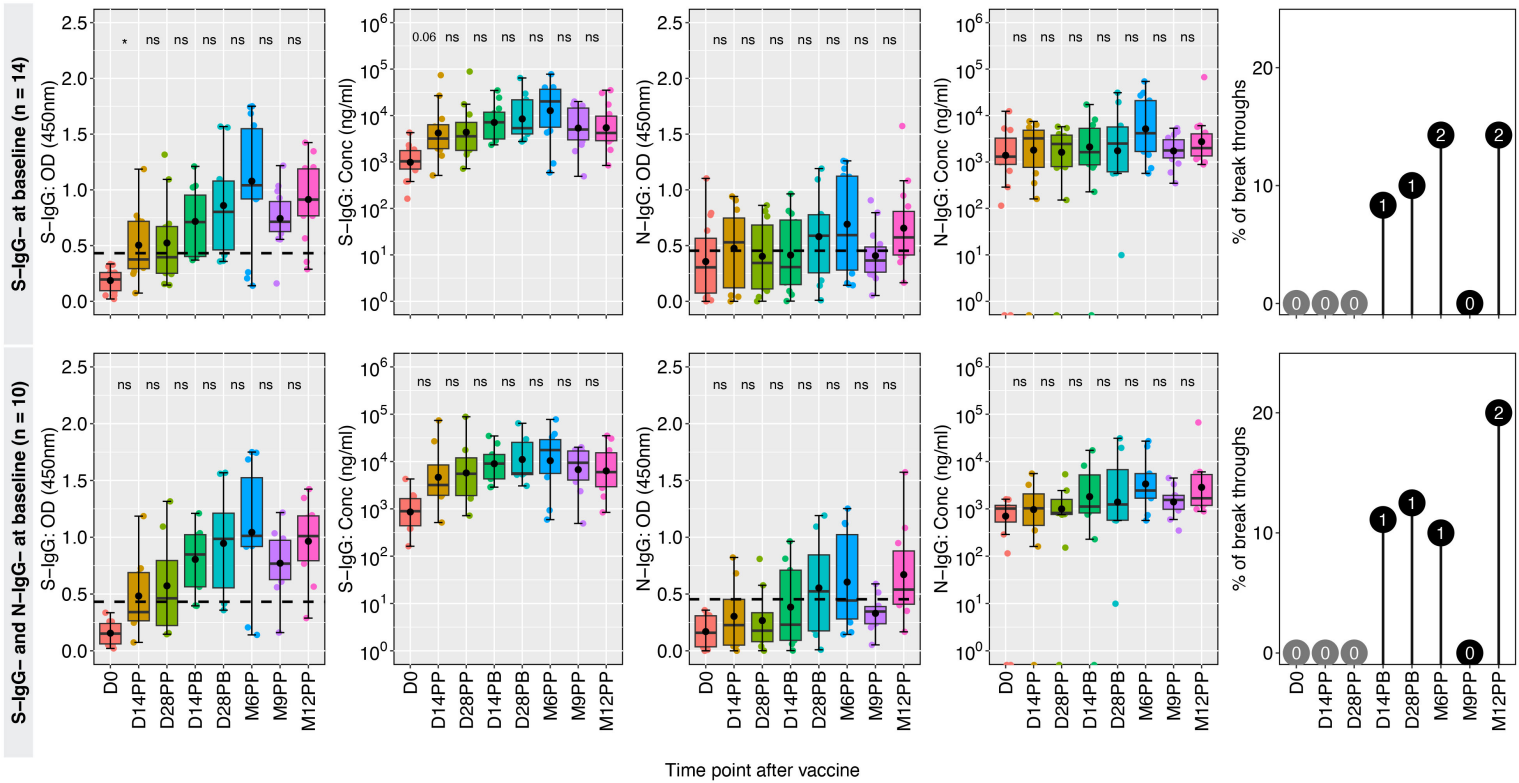
CoronaVac-induced immunity among the previously naive group. **Figure 5** showcases the temporal changes in antibody responses among 14 individuals who lacked seropositivity for S-IgG antibodies and 10 individuals who lacked seropositivity for both S-IgG and N-IgG antibodies at baseline (D0), indicating a presumed lack of prior exposure to the antigen before vaccination. Box plots represent the summarized data of spike-directed IgG antibody OD values and concentrations, including the means (represented by dots), medians (horizontal lines), and interquartile ranges (top and bottom of the box). Additional boxplots display the OD values for S-IgA and N-IgG antibodies. Infections before the full vaccine dose are highlighted in pink, while breakthroughs defined as infections within two weeks of completing the full dose are indicated in blue. The number of infections is indicated within the corresponding circles, providing insights into the infection patterns and vaccine efficacy. A p-value greater than 0.05 was deemed insignificant (indicated as “ns”; not significant). Statistical significance is denoted by “*” representing a p-value less than 0.05.

In a group of 14 participants who were spike antibody naive at baseline, the S-IgG seropositivity rate steadily increased from 0% to 42% at 14 and 28 days post-primary vaccination (D14PP and D28PP). Subsequently, seropositivity rose, reaching 68% and 70% at 14- and 28-days post-booster, respectively. By the sixth month following the initial vaccination, 78% of vaccinated individuals demonstrated seropositivity, which increased to a peak of 93% after nine months and maintained a level of approximately 90% by the twelfth month, alongside a rise in S-IgG antibody concentration. We observed a significantly higher incidence of breakthrough infections (42%) in antibody-naive participants compared to those with hybrid immunity (13%), possibly contributing to the persistence of spike-directed antibodies. The findings also revealed consistently low levels of S-IgM and S-IgA antibodies. All breakthrough infections were asymptomatic, indicating the protective benefits conferred to the vaccinated individuals. Among the 12 individuals lacking both S-IgG and N-IgG antibodies at baseline, antibody levels peaked at six months but subsequently declined, summarized in **Figure 5**. In S-IgG naive individuals, seropositivity for Spike IgG gradually increased from baseline to 37.5% and 50% at 14 and 28 days after the first dose and further rose to 78% 14 days after the booster dose, reaching 80% after six months and eventually reaching 90% by 9 and 12 months (**Table 2**). There was a minimal increase in fold changes between any two-time points. Specifically, the OD responses showed a doubling effect from D0 to D14PP. Following the administration of the second dose, the S-IgG levels experienced almost no change (fold change of 1.13). However, no substantial fold changes were observed after the second dose, as the fold change remained close to 1 for all antibodies. Persistence in vulnerability was observed in a subset of individuals as breakthrough infections emerged at different time intervals following primary vaccination, including 14 days, 28 days, six months, nine months, and 12 months, with one subject experiencing infection at both days 14 post-boost and the 12-month mark. This study offers valuable insights into the dynamics of antibodies among individuals who were initially negative for anti-spike antibodies but received the CoronaVac vaccination. The findings demonstrate an initial rise in S-IgG antibodies, followed by a decrease after 6 months.

**Table 2:** Seroconversion Rates Over Time for 10 Subjects Identified as Spike and Nucleoprotein Seronegative (S-IgG and N-IgG) at Baseline.

Overall Cohort	D0 (n = 60)	D14PP (n = 54)	D28PP (n = 54)	D14PB (n = 49)	D28PB (n = 49)	M6PP (n = 54)	M9PP (n = 52)	M12PP (n = 51)
S-IgG- and N-IgG- at baseline	10	8	8	9	8	10	10	10
**S-IgG**	0 (0.00%)	3 (37.5%)	4 (50.0%)	7 (77.8%)	6 (75.0%)	8 (80.0%)	9 (90.0%)	9 (90.0%)
**S-IgM**	1 (10.0%)	3 (37.5%)	0 (0.00%)	1 (11.1%)	3 (37.5%)	1 (10.0%)	0 (0.00%)	0 (0.00%)
**S-IgA**	1 (10.0%)	3 (37.5%)	2 (25.0%)	2 (22.2%)	2 (25.0%)	6 (60.0%)	6 (60.0%)	6 (60.0%)
**N-IgG**	0 (0.00%)	2 (25.0%)	2 (25.0%)	3 (33.3%)	5 (62.5%)	4 (40.0%)	2 (20.0%)	6 (60.0%)
**N-IgM**	1 (10.0%)	0 (0.00%)	0 (0.00%)	0 (0.00%)	0 (0.00%)	0 (0.00%)	1 (10.0%)	2 (20.0%)

**Table 2** presents the counts (n) and corresponding proportions (percentages) of subjects exhibiting seropositivity for IgG, IgM, and IgA antibody isotypes against both the spike and nucleoprotein at each respective time point. The displayed data pertains explicitly to a subset of subjects initially identified as seronegative for antibodies targeting spike and nucleoprotein at baseline (denoted as baseline S-IgG- and N-IgG-). The table also provides the total number of subjects from the main cohort and those classified as S-IgG- and N-IgG- at baseline.

The counterintuitive trend observed, where individuals lacking seropositivity at baseline (D0) report fewer infections than presented in **Figure 4**, warrants careful interpretation. The limited sample size in our study may not offer the statistical strength to conclusively determine the variation in infection rates between seropositive and seronegative groups. Limited datasets can sometimes produce statistical outliers. Hence, drawing definitive conclusions based on this observation should be approached with caution.

## Discussion

The immune response of African populations to the CoronaVac inactivated vaccine, which was widely administered during the COVID-19 vaccination campaigns, remains largely unknown. We investigated the dynamics of antibody responses targeting the SARS-CoV-2 spike and nucleoproteins after CoronaVac vaccination, comparing real-life cohorts with different pre-existing IgG statuses. Our primary findings shed light on the vaccine’s effectiveness in eliciting immune responses in different cohorts, revealing distinct patterns of antibody responses and breakthrough infections. High levels of prior and subsequent spike-specific IgG antibody cross-reactivity were noted throughout the follow-up period in a mixed cohort of varying IgG seropositivity. The study revealed consistently high and stable seropositivity rates for spike-IgG antibodies after CoronaVac vaccination of a largely prior exposed population, indicating the effective stimulation of long-term immunity against SARS-CoV-2. In contrast, seropositivity rates of spike-IgM antibodies were limited, indicating a suboptimal IgM response following vaccination of an exposed population. There was a decline in spike-IgM positivity frequency over time, while spike-IgA antibody levels remained moderate and stable throughout the study period. The fold change analysis across pairwise time points consistently approached 1 for both S- and N- antibody responses, indicating a relatively stable and comparable antibody profile over time. These findings suggest that the vaccine-induced antibody responses remain relatively constant beyond the initial boost and do not show significant fluctuations. Coinciding breakthroughs could contribute to spike-directed IgG persistence.

We noted significant differences in individuals with prior IgG positivity compared to those with IgG negativity at baseline. Those with prior IgG showed consistently strong and sustained spike-IgG antibody seropositivity ranging from 93% to 100%, indicating a robust immune response in those with prior spike protein exposure, likely due to hybrid immunity as described for other vaccines (14, 15). Individuals who initially tested negative for spike-IgG antibodies demonstrated a gradual and steady increase in seropositivity rates following vaccination, indicating the vaccine’s ability to elicit an immune response even in previously unexposed individuals. These data show that the baseline IgG status influenced antibody response to CoronaVac vaccination, underscoring the importance of considering prior exposure in determining vaccine efficacy. The CoronaVac vaccine primarily induced a robust IgG response, while the IgM and IgA responses appeared to have a lesser role in long-term immunity against SARS-CoV-2.

Consistent with others (16), anti-spike IgG antibodies persisted but at moderately low levels following CoronaVac vaccination. Vaccination resulted in substantial increase in the S-IgG levels, surpassing those observed in participants without prior exposure, aligning with the concept that hybrid immunity provides a more robust immune response, described in other studies (17–19). This study adds to others that showed CoronaVac’s ability to generate potent antibodies against SARS-CoV-2 (17, 19), underscoring the importance of boosting, providing evidence of CoronaVac ability to elicit long-lasting anti-spike antibodies against SARS-CoV-2. The IgG response induced by CoronaVac remained stable until six months, when it began to decline. This pattern aligns with this population’s previously observed response to ChAdOx1-S (AstraZeneca—University of Oxford) vaccination. Elevated antibody concentrations remained detectable at 12 months, regardless of baseline exposure status, mirroring the patterns observed with ChAdOx1-S in this demographic (15). The suboptimal IgM response in previously unexposed individuals agrees with inactivated vaccines being more associated with IgG dominance than IgA (20) and the decline in IgM levels following antigenic exposure (7). Also, the moderate IgA response observed here is consistent with the notion that IgA antibodies have a more limited contribution to long-term immunity than IgG antibodies (21, 22). The delayed peaking of antibody responses aligns with others that showed a gradual but sustained increase in antibody levels in response to the CoronaVac vaccine over time (20), as opposed to the rapid development and peaking of antibodies in response to natural infections (21, 23). The declining levels of antibodies six months after the initial dose aligns with previously observed trends elsewhere (24). Given that this is a whole particle based vaccine, it is crucial to consider measuring immune responses to other structural targets of the virus, such as the envelope and membrane, as these have been identified to contribute to the induced immunity (25–27), although they were not assessed in this study.

Individuals without pre-existing cross-reactive spike-directed IgG antibodies had lower vaccine-induced anti-spike antibody levels and a four-fold higher risk of breakthrough infections compared to those with prior spike-IgG positivity, consistent with reports of weaker immune responses in antibody naive populations (28). The baseline spike-negative group had higher breakthrough rates than those with prior exposure, highlighting their increased vulnerability to breakthrough infections. This is consistent with previous studies showing that prior seropositivity before vaccination leads to highly potent antibodies with broader neutralizing activity (29–31). Understanding the impact of pre-existing antigen exposure on the immune system is crucial, as it reveals the potential for enhanced antibody responses and improved functional capabilities. Incorporating this knowledge into vaccine strategies becomes imperative, as it highlights the importance of considering pre-existing seropositivity to optimize vaccination approaches. By recognizing and accounting for prior antigenic exposure, we can develop more effective vaccines that harness the full potential of the immune system, ultimately leading to better protection against infectious diseases. We found a breakthrough rate of 20%, which aligns closely with the documented efficacy of the CoronaVac vaccine in preventing symptomatic cases at 65.9% (1, 32). In participants who tested negative for antibodies at baseline, an intriguing, presumed breakthrough rate of 42% was detected during the study. Remarkably, despite testing positive for the infection, all these breakthrough cases reported no symptoms or signs of illness. Our findings are consistent with a comprehensive clinical trial conducted in Chile, which proved that the CoronaVac vaccine prevents severe disease (32).

This study provides valuable real-life insights into the long-term dynamics of antibody responses to CoronaVac vaccination in the sub-Saharan setting. The strengths of this data include a longitudinal analysis that spans up to 12 months, stratification of the cohort by prior spike-IgG seropositivity status, and the consistent monitoring of multiple antibody isotypes (S-IgG, S-IgM, S-IgA, N-IgG, and N-IgM) over time. However, it is essential to note some limitations to consider when interpreting the data. First, the relatively small sample size of 60 participants, further stratified by prior exposure status may restrict generalizability of the study. Elsewhere, individuals who received inactivated vaccines but did not have prior infections reduced their protection against Omicron and its variants, while those with previous infections exhibit robust neutralizing antibodies and memory B cells, indicating stronger immunity. Encouragingly, T-cell responses appeared unaffected, underscoring T-cell-mediated cellular immunity’s potential to provide substantial protection (33). We did not assess cellular immune responses or T-cell immunity, which are important for adequate protection against viral infections. Lastly, significant variations in population demographics (34), methodologies (35), and timing of sample collection should be taken into consideration as potential factors contributing to these differences, as previous studies have indicated that females may exhibit higher antibody responses compared to males (36). We did not stratify the analyses further by gender. Given the frequencies of baseline exposure, our primary objective was to categorize participants based on prior exposure, leaving the sample size insufficient to enable further stratification by gender. Furthermore, as this cohort predominantly consisted of youths, any stratification by age would not provide a substantial sample size for meaningful comparisons. Future studies should address these limitations to provide a more comprehensive understanding of the vaccine’s effectiveness and inform public health strategies.

Overall, this study offers significant insights into the longevity of spike-directed IgG antibodies post-CoronaVac vaccination, revealing enduring hybrid immune responses with sustained levels and high seropositivity rates over 12 months, yet highlighting the need for continuous monitoring due to breakthrough infections in some individuals. Robust and long-lasting antibody responses were observed in individuals with prior virus exposure and higher baseline antibody levels. In comparison, those with lower pre-vaccination antibody levels displayed increased vulnerability to breakthrough infections. Despite breakthrough infections occurring in some individuals, no symptoms were reported, highlighting the critical role of vaccination in providing protection.

## Data availability statement

The raw data supporting the conclusions of this article will be made available by the authors, without undue reservation.

## Ethics statement

The studies involving humans were approved by the Uganda Virus Research Institute (UVRI REC Ref: GC/127/20/04/773) and the Uganda National Council for Science and Technology (UNCST Ref: HS637ES). The studies were conducted in accordance with the local legislation and institutional requirements. The participants provided their written informed consent to participate in this study.

## Author contributions

JaS: Methodology, Project administration, Writing – review & editing, Data curation. CB: Data curation, Methodology, Project administration, Writing – review & editing. VA: Data curation, Methodology, Writing – review & editing, Formal analysis, Validation, Writing – original draft. JK: Data curation, Methodology, Writing – review & editing. GOl: Data curation, Methodology, Writing – review & editing, Formal analysis, Visualization. CA: Data curation, Methodology, Writing – review & editing, Validation. LK: Data curation, Methodology, Validation, Writing – review & editing, Visualization. GOd: Data curation, Methodology, Writing – review & editing. PE: Data curation, Methodology, Writing – review & editing, Project administration. SO: Data curation, Methodology, Writing – review & editing, Validation. MM: Writing – review & editing, Resources, Supervision. PK: Methodology, Writing – review & editing, Formal analysis, Supervision. JeS: Formal analysis, Methodology, Supervision, Writing – review & editing, Conceptualization, Funding acquisition, Investigation, Project administration, Resources, Writing – original draft.

## Group member of the COVID-19 immunoprofiling team

Angella Namuyanja, Susan Mugaba, Ivan Ssali, Arthur Kalyebi Watelo, Hellen Nantambi, Ben Gombe, Betty Auma.
